# Smart materials strategy for vascular challenges targeting in-stent restenosis: a critical review

**DOI:** 10.1093/rb/rbaf020

**Published:** 2025-03-24

**Authors:** Kai Zhang, Wenzhao Liang, Xiao-Bo Chen, Jing Mang

**Affiliations:** Department of Geriatrics and General Practice, China-Japan Union Hospital of Jilin University, Changchun 130033, China; Department of Geriatrics and General Practice, China-Japan Union Hospital of Jilin University, Changchun 130033, China; School of Engineering, RMIT University, Melbourne, VIC 3000, Australia; Department of Neurology, China-Japan Union Hospital of Jilin University, Changchun 130033, China

**Keywords:** in-stent restenosis, smart materials, targeted responses, triggers

## Abstract

In-stent restenosis (ISR) presents a major challenge in vascular disease management, often leading to complications and repeated interventions. This review article explores the potential of existing smart materials strategies in addressing ISR, emphasizing advancements in materials science and biomedical engineering. We focus on innovative solutions such as bioactive coatings and responsive polymers that offer targeted responses to ISR-related internal and external triggers. These smart materials can dynamically adapt to the physiological conditions within blood vessels, responding in real time to various stimuli such as pH, oxidative stress and temperature. Moreover, we discuss preclinical progress and translational challenges associated with these materials as they move toward clinical applications. The review highlights the importance of controlled drug release and the need for materials that can degrade appropriately to minimize adverse effects. This work aims to identify critical research gaps and provide guidance to encourage interdisciplinary efforts to advance the development of smart stent technologies. Ultimately, the goal is to improve patient outcomes in vascular interventions by leveraging the capabilities of intelligent biomaterials to enhance ISR management and ensure better long-term efficacy and safety in-stent applications.

## Graphical abstract

**Figure:**
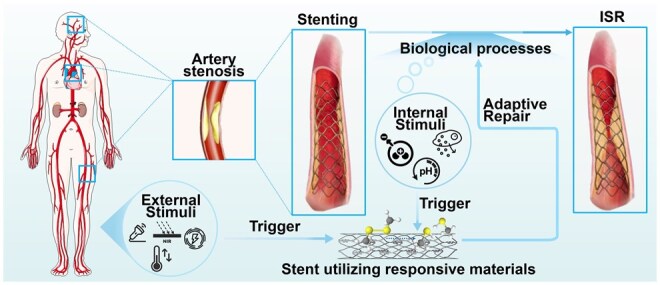

## Introduction

Vascular diseases, in particular those affecting coronary, cerebral and peripheral arteries, are major contributors to global morbidity and mortality, often leading to severe complications such as stroke, myocardial infarction and limb ischemia [1–3]. Stent technology plays a critical role in managing those conditions by reopening narrowed arteries and restoring blood flow [4, 5]. However, a long-lasting challenge associated with stent implantation is in-stent restenosis (ISR), which is characteristic of excessive tissue regrowth that re-narrows or obstructs artery after implantation [6, 7]. The first generation of bare-metal stents provides mechanical support to maintain arteries open but commonly leads to ISR [8]. This phenomenon is primarily attributed to vascular smooth muscle cell (VSMC) proliferation, neointimal hyperplasia, extracellular matrix (ECM) remodeling and other related processes, ultimately leading to the re-narrowing of the artery [9].

To address such critical challenges, drug-eluting stents (DES) were developed to deliver antiproliferative drugs to the impacted sites and inhibit cell proliferation [10]. Though DES reduces restenosis rates, new challenges embark, particularly a lack of controlled drug release, which often leads to an initial burst release of drugs [11, 12]. Such wild drug-release profiles can result in poor long-term sustainability and a variety of adverse effects, including late thrombosis and chronic inflammation given the permanent presence of stent [13, 14]. To tackle the issues associated with permanent implants, bioresorbable stents are designed and introduced to gradually degrade after fulfilling their purposes [15]. Despite their potential, controllable degradation rates and drug-release profiles in the dynamic and varied conditions of vascular system remains a significant challenge, leading to ongoing concerns about ISR [15, 16].

To advance current stent designs, a great deal of effort focuses on the development of intelligent biomaterials—stimuli-responsive materials—that can dynamically adapt to the changing physiological conditions within blood vessels [17]. These materials can respond in real time to various endogenous or external stimuli, such as pH, oxidative stress, temperature, etc. Such adaptability holds a great promise for maximizing drug-release precision and biocompatibility, potentially providing more effective solutions to the challenges posed by ISR [18].

As such, this review article aims to explore feasible ways to tailor a large variety of intelligent biomaterials to target different processes involved in ISR. Various types of material’s responsive mechanisms to these processes are examined, along with the developmental challenges they encounter. In addition, we discuss the primary obstacles toward clinical applications and potential solutions from the perspectives of both clinicians and engineers. By understanding these dynamics, it is expected to provide insightful guidance to the design and implementation of smart materials to yield effective ISR management and satisfactory clinical performance.

## Smart stent materials responding to internal stimuli

### pH-responsive materials for ISR

#### Mechanisms of pH changes in ISR

**Figure 1.**
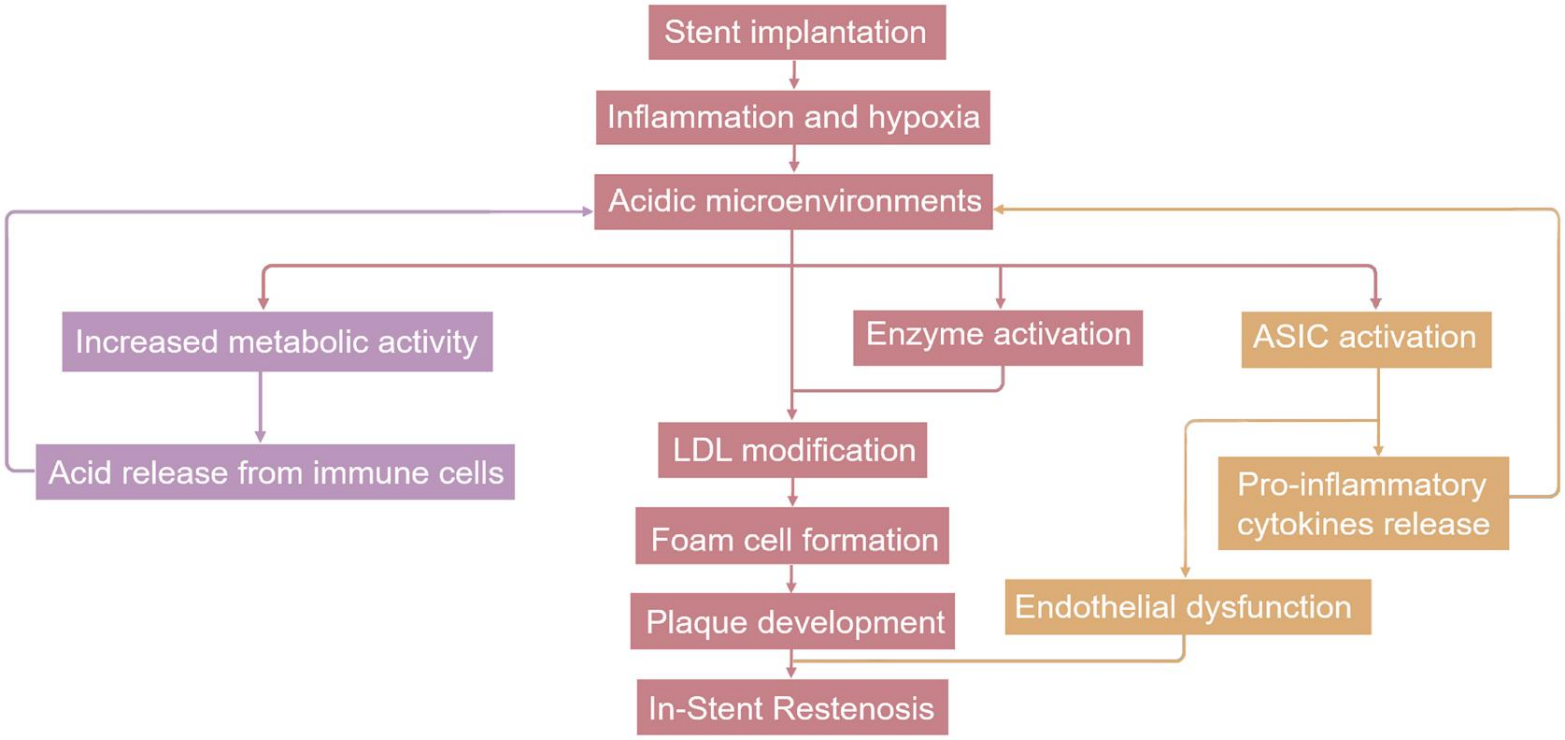
Pathways leading to in-stent restenosis via pH changes in vascular microenvironments.

During ISR, mechanical damage from stent placement creates an acidic microenvironment (pH 6.0–7.0), which exacerbates inflammation and promotes atherosclerosis [19–21], as depicted in Figure 1. In such acidic settings, acid-sensing ion channels become activated, further damaging endothelial cells (ECs), enhancing macrophage uptake of low-density lipoprotein, and leading to foam cell accumulation. This accumulation contributes to lipid core development within atherosclerotic plaques, accelerating their progression [20–22]. Additionally, pH shifts influence the activity of enzymes such as matrix metalloproteinases (MMPs), which degrade ECM components like collagen and elastin. These changes facilitate VSMC migration and proliferation, further driving neointimal hyperplasia and ECM remodeling. The acidic conditions also increase reactive oxygen species (ROS) production, triggering oxidative stress. ROS stimulate ECM degradation and enzyme activation, such as the upregulation of MMPs, which further accelerates vascular remodeling and inflammation. Together, these factors form a cascading reaction. Thus, pH changes act as early and broad triggers in ISR, initiating a network of interrelated oxidative stress, enzyme activation and inflammatory responses [20].

#### pH-responsive drug delivery systems

**Figure 2.**
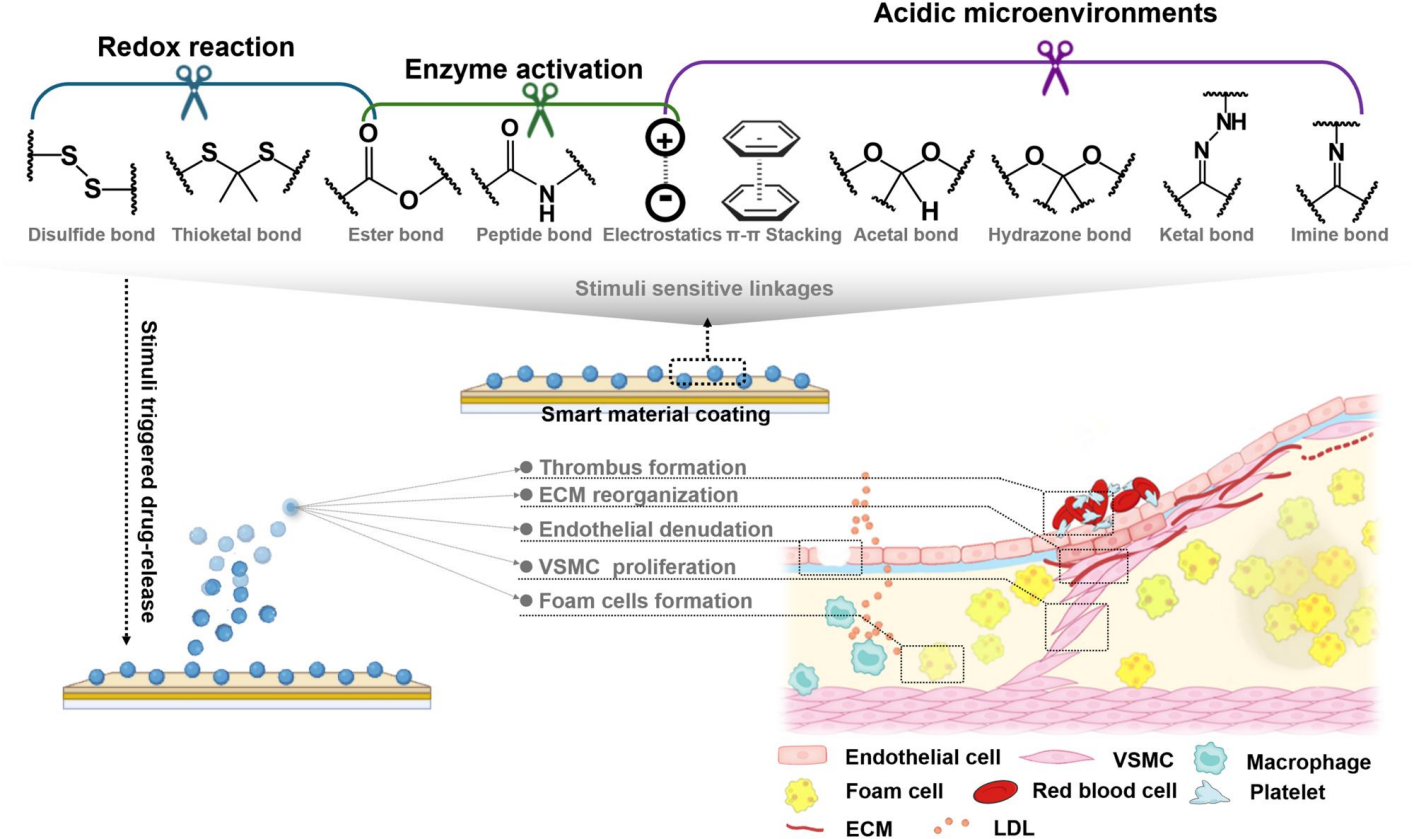
Stimuli-responsive linkages in smart materials for targeted healing of in-stent restenosis.

Drug delivery systems with a pH-responsive switch originated in cancer therapy to release drugs in acidic environments [23]. Such working mechanisms are triggered by breaking pH-sensitive bonds. Given a large number of anti-ISR drugs overlapping with cancer treatments, such systems show great promise for targeted ISR therapy, sparking interest in their applications in vascular stents (Figure 2).

##### Electrostatic interaction

In pH-responsive drug delivery systems, electrostatic interactions are crucial for drug release [24, 25]. For example, poly(styrenesulfonate) (PSS)-modified layered double hydroxide (LDH) films remain stable in neutral or alkaline conditions due to interactions between the sulfonate groups (–${\text{SO}}_{3}^{-}$) in PSS and metal ions (Ni^2+^ and Ti^4+^) on the LDH surface. In acidic environments, protonation of these sulfonate groups weakens the interactions, destabilizing the membrane and releasing drugs such as doxorubicin (DOX) [26]. Similarly, PSS contributes to layer-by-layer polyelectrolyte coatings on nanoporous anodic alumina, where protonation of poly(allylamine hydrochloride) enhances layer repulsion, causing swelling and promoting controlled drug release, with up to 90% of the drug released within 24 hours at pH 5.2, compared to only 30–40% at pH 7.4 [27]. In contrast, DES often face challenges with uncontrolled burst release, especially in the initial hours. Protonation of PSS-modified hollow mesoporous silica nanoparticles, octenylsuccinic anhydride-modified chitosan (OSA-CS) nanoparticles and methoxy-poly(ethylene glycol) (mPEG)-poly (ε-caprolactone) (PCL)-poly(diethylaminoethyl methacrylate) (PDEA) micelles trigger swelling, facilitating drug release [28–30]. For example, curcumin (CUR)-OSA-CS and quercetin (QUE)-OSA-CS show pH-dependent release: at pH 7.4, their release is under 50% within 48 hours, but at pH 6.0, they release about 90% within 48 hours, indicating the potential for more targeted drug delivery compared to the typical release profiles of DES [28].

##### π–π Stacking

pH-responsive PCL/functionalized carbon nano-onions (f-CNOs) nanofibers release drugs by weakening the π–π stacking interactions and hydrogen bonds between the f-CNOs and the encapsulated drugs in acidic environments. This weakening increases the nanofiber’s porosity, allowing for controlled drug release. For example, the release of DOX from PCL/f-CNOs composite fibers reached 87% at pH 6.5 and nearly 99% at pH 5.0 over 15 days. In neutral environments, the strong interactions between f-CNOs and the PCL matrix stabilize the nanofiber structure, securely encapsulating drugs such as DOX within the fibers. In acidic environments, the disruption of these interactions significantly increases the porosity of the fibers, accelerating drug release [31].

##### Chemical bonds

pH-responsive drug delivery systems often rely on chemical bonds that undergo hydrolysis or cleavage in acidic environments to enable controlled drug release. For instance, benzoyl imine linkages in pH-sensitive micelles release over 90% of paclitaxel (PTX) within 48 hours at pH 5.5, compared to just 50% at pH 7.4 [32]. This controlled release profile contrasts with the initial burst release seen in DES, offering greater stability under physiological conditions. Similarly, ketal linkages hydrolyze more efficiently at low pH, releasing therapeutic agents like superoxide dismutase at sites of inflammation [33]. Acetal bonds also contribute to pH-responsive release in dual-responsive systems for vascular inflammation. For example, pH/ROS dual-responsive nanoparticles (defined as AOCD NPs) composed of acid-sensitive derivative of β-cyclodextrin and ROS-sensitive derivative of β-cyclodextrin exhibit excellent biocompatibility, with no hemolysis observed even at concentrations as high as 2 mg/ml and show no significant toxicity at doses as high as 1000 mg/kg in rat studies [34]. Under acidic conditions, hydrazone bonds in hyaluronic acid-based nanoparticles enable targeted acid-triggered release, enhancing drug delivery in inflammatory and atherosclerotic contexts. At pH 7.4, the release rate of all-trans retinoic acid (ATR) remains below 10% within 48 hours, while rapamycin (RAP) shows relatively slow release. At lower pH (e.g. pH 6.5 and pH 5.2), both ATR and RAP release rates significantly increase, with ATR reaching 80.3% at pH 5.2 and RAP reaching 94.9% [35]. Metal-coordination complexes, such as iron-tannic acid assemblies, dissociate under acidic conditions to release antioxidants, demonstrating promising pH-sensitive properties for therapeutic applications [36]. Likewise, mesoporous silica nanoparticles functionalized with phenanthroline exhibit pH-triggered drug release, with copper-phenanthroline coordination bonds dissociating at pH 5 to release curcumin [37]. Additionally, mesoporous nanoplatforms with pH-sensitive dynamic covalent bonds facilitate controlled release via protonation, offering improved targeted therapy and minimizing side effects [38].

#### Self-healing pH-responsive coatings

pH-responsive coatings for magnesium-based biodegradable stents and implants are vital for controlling material degradation, promoting self-healing and supporting tissue regeneration [39, 40]. These coatings use pH-triggered mechanisms to release ions or inhibitors that regulate degradation rates and maintain mechanical integrity. Similarly, pH-responsive nanocontainers with corrosion inhibitors like 2-mercaptobenzothiazole release these compounds in acidic environments, slowing magnesium corrosion and preserving structural integrity [41]. Chitosan multilayer coatings for magnesium alloys leverage chitosan swelling in alkaline conditions to release ceria nanoparticles, which inhibit corrosion and fill cracks, ensuring controlled degradation and material integrity [42].

#### Practical challenges

Despite continuous advancements in pH-responsive materials, their clinical application in managing ISR is hindered by the variability of local pH following stent implantation. Factors such as inflammation, stress and coating properties lead to fluctuations in the microenvironment, complicating the consistent release of therapeutic agents. Additionally, the lack of sufficient preclinical and clinical data to guide optimization exacerbates these challenges [43, 44]. Additionally, long-term biocompatibility and mechanical stability present critical hurdles; prolonged exposure to these materials often induces inflammation and undermines stent integrity, compromising therapeutic outcomes [45]. To overcome these challenges, integrating multi-responsive systems that combine pH with other ISR triggers may enhance precision, while real-time pH monitoring can improve responsiveness. Layer-by-layer structures may offer better control release rates, reducing initial bursts [27]. Meanwhile, hybrid materials and bioinert coatings can boost stability and biocompatibility [41].

### Redox-responsive materials

#### Mechanisms of redox reactions in ISR

Redox reactions, particularly those mediated by ROS, are critical drivers of oxidative stress during ISR. Under physiological conditions, ROS—including superoxide anions ($O_{2}^{-}$), hydrogen peroxide (H_2_O_2_) and hydroxyl radicals (OH·)—are integral to cellular signaling pathways and immune defense mechanisms [46, 47]. However, tissue damage and inflammation following stent implantation result in pathological elevations in ROS levels, overwhelming antioxidant defenses such as glutathione (GSH) and glutathione peroxidase (GPx) and initiating sustained oxidative stress[48, 49]. This imbalance disrupts endothelial function, promotes VSMC proliferation and accelerates ECM deposition, ultimately contributing to neointimal hyperplasia [50].

The deleterious effects of excessive ROS extend beyond direct cellular injury, as oxidative stress amplifies other pathological processes. For example, heightened ROS activity induces the expression of MMPs, which degrade ECM components and drive maladaptive vascular remodeling [48, 49]. These interconnected pathways underscore the multifaceted role of oxidative stress in ISR progression, highlighting its potential as a therapeutic target.

#### ROS-responsive materials

ROS-responsive materials are engineered to degrade in environments with elevated levels of ROS, enabling localized and targeted drug release. The functionality of these materials relies on the incorporation of ROS-sensitive chemical bonds, which undergo oxidative cleavage to release therapeutic agents in a controlled manner (Figure 2, Table 1).

**Table 1.** Impact of redox-responsive materials on ISR development

Material (stimulus)	Responsive mechanism	Released agents	Impact	Ref(s)
ROS-responsive (H_2_O_2_, OH·)	Disulfide bonds oxidize, breaking the crosslinked structure	Pitavastatin, Allicin, H_2_S	Inhibit VSMC proliferation, reduces inflammation	[51, 52]
ROS-responsive (H_2_O_2_)	Thioketal bonds selectively degrade in high ROS environments	Dexamethasone	Anti-inflammatory effect, targeted release	[53, 54]
ROS-responsive (H_2_O_2_)	Boronic ester bonds oxidize, releasing TEMPO and exosomes	TEMPO, Exosomes	Reduces oxidative stress, promotes endothelial repair	[57, 58]
GSH-responsive (high GSH)	Disulfide bonds reduce in high GSH environments, releasing encapsulated agents like VEGF	VEGF	Endothelial repair, VSMC inhibition	[59]
GSH-responsive (high GSH)	Simulates GPx catalysis, producing NO from GSNO in GSH-rich environments	NO	Vasodilation, anti-inflammatory, VSMC inhibition	[60, 61]
Dual-responsive (ROS, GSH)	Disulfide bonds degrade via ROS or GSH, enabling dynamic release	NO	VSMC inhibition, endothelial healing	[61, 65]

GSNO, S-nitrosoglutathione; TEMPO, 2,2,6,6-tetramylpiper-idine-n-oxyl.

##### Disulfide bond

Disulfide bonds are frequently employed in ROS-responsive systems, including coatings like pitavastatin calcium and allicin-H_2_S hydrogels. In oxidative microenvironments, disulfide bonds are oxidized to sulfinic or sulfonic acids, destabilizing the material matrix and triggering drug release [51, 52]. This mechanism ensures precise drug delivery to sites of elevated oxidative stress, where agents such as pitavastatin effectively inhibit VSMC proliferation and mitigate restenosis progression [51]. Specifically, The EpigallocatechinGallate (EGCG)-cystamine (Cys)-Pitavastatin calcium (Pi) coating released 31.5% of its drug load in 12 hours in a 1 mM H_2_O_2_ solution, with slightly higher release in 200 μM H_2_O_2_ compared to phosphate-buffered saline (PBS). In rabbit arteries, it caused minimal neointimal thickening (3.45–3.71% after 3 months), while EGCG-hexamethylenediamine (HMD)-Pi caused a significantly greater increase [51]. Similarly, the catechol hyaluronic acid (C-HA)-Cys-Allicin coating released 25.45 μmol/l of H_2_S in 200 μM H_2_O_2_ after 12 hours, significantly more than in PBS (4.12 μmol/l). In an *in vitro* thrombosis assay, it showed the lowest thrombus weight (0.16 mg) and 15.7% thrombus occlusion [52].

##### Thioketal bond

Thioketal (TK) bonds remain stable under physiological conditions but degrade selectively in ROS-rich environments, enabling precise drug release while preventing premature leakage. Dexamethasone-loaded systems leverage thioketal bonds to target oxidative stress regions, improving therapeutic outcomes [53, 54]. For example, in the presence of 0.2 mM H_2_O_2_, dexamethasone (Dex) released 50.45% over 30 days, with a higher release of 78.95% at 1 mM H_2_O_2_. In contrast, in the absence of H_2_O_2_, 95.1% of Dex remained in the “Antifouling plus” coating (defined as D-MAP coating), constructed from Dopamine-Polyethyleneimine-Thioketal-Dex and Poly(2-methacryloyloxyethyl phosphorylcholine-co-lauryl methacrylate-co-methacrylic acid), after 30 days. *In vivo*, the D-MAP group showed lower stenosis rates (23.93% at 1 month and 25.36% at 3 months) compared to PLA and MAP groups (30.93% and 34.21% at 1 month, respectively) [53]. Similarly, mPEG-TK-PCL nanomicelles encapsulate DOX with structural stability, releasing the drug upon ROS-mediated cleavage [55]. Thioketal-based polymers, such as poly(thioketal) (PTK)-urethane (UR), also regulate scaffold degradation rates in oxidative microenvironments, supporting controlled tissue regeneration [56].

##### Ester bond

Boronic ester bonds react to ROS by undergoing oxidation in oxidative environments, leading to the release of bioactive agents such as 2,2,6,6-tetramylpiper-idine-n-oxyl. These materials, commonly used in exosome coatings and heparin-mimicking hydrogels, help alleviate oxidative stress and promote endothelial repair [57, 58].

#### GSH-responsive materials

GSH-responsive materials leverage elevated GSH levels in ISR lesions, particularly in later stages. Disulfide bonds degrade in GSH-rich environments, breaking into thiol groups (–SH) and releasing encapsulated drugs like vascular endothelial growth factor (VEGF), which promote endothelial healing [59]. Disulfide bonds also play a dual role by mimicking GPx activity, catalyzing the release of nitric oxide (NO) from S-nitrosoglutathione in GSH-rich conditions. This promotes vasodilation, reduces VSMC proliferation and helps prevent ISR [60, 61]. Meanwhile, biomimetic nanoparticle coating (BMC) coatings show sustained NO release, with an initial rate of 4.2 × 10^(−10)^ mol·cm^(−2)^·min^(−1)^, maintaining 55.05% after 90 days. *In vivo*, BMC coatings reduced neointimal thickness to 132.1 ± 13.0 μm at 6 months, compared to 380.1 ± 28.8 μm for bare stents [60]. In similar designs, β-cyclodextrin-linked S-nitrosothiols release NO in response to intracellular GSH, supporting endothelial function and selectively inhibiting VSMC proliferation in ISR lesions [62].

#### Dual ROS- and GSH-responsive materials

Dual-responsive materials integrate ROS- and GSH-sensitive bonds, enabling precise drug release tailored to the local oxidative and reductive microenvironments [63]. An example of such materials is EGCG-cystamine coatings, where cystamine, through disulfide bonds that degrade under oxidative conditions, suppresses VSMC proliferation and promotes endothelial healing in ISR [64]. Similarly, pitavastatin-NO delivery systems employ redox-responsive mechanisms to release NO under ROS-mediated conditions and catalyze further NO generation through GSH interaction, enhancing endothelial regeneration and inhibiting neointimal hyperplasia [61]. Additionally, tellurium-functionalized polyurethane coatings represent a promising dual-responsive platform. By mimicking glutathione peroxidase activity, these coatings dynamically release NO in the presence of both ROS and GSH, demonstrating potent antithrombosis, anti-inflammatory and antihyperplasia effects at ISR sites [65].

#### Practical challenges

The clinical application of redox-responsive materials in ISR management faces challenges stemming from the dynamic oxidative and reductive microenvironments. After stent implantation, ROS levels surge, driving inflammation and oxidative damage, but subsequently decline as the lesion stabilizes, which can undermine the sustained efficacy of ROS-responsive systems and result in inconsistent drug release. Concurrently, elevated GSH levels in advanced ISR stages, critical for cellular repair, may prematurely trigger GSH-responsive materials, disrupting controlled release and limiting therapeutic effectiveness [66]. To address these challenges, dual-responsive systems capable of adapting to both oxidative surges and gradual GSH increases offer a promising solution. Such systems could synchronize drug release with the dynamic oxidative-reductive progression of ISR, enhancing therapeutic precision. Moreover, tellurium-based polymer coatings, by mimicking glutathione peroxidase activity, neutralize residual ROS and mitigate inflammation, improving biocompatibility and reducing post-implantation complications [65]. While redox-responsive drug delivery systems enable targeted release, the biocompatibility of degradation byproducts remains a concern for long-term use. For instance, in nucleic acid-coated vascular stents with polycation-based delivery systems, degradation can release free cationic polymers, which may cause hemolysis and secondary thrombosis, highlighting the need for comprehensive biocompatibility assessments of both acute hematological responses and long-term compatibility [59].

### Enzyme-responsive materials

#### Mechanisms of enzyme change in ISR

Enzymatic activity, particularly from MMPs, plays a crucial role in ISR by driving ECM degradation and vascular remodeling. MMPs, including MMP-2 and MMP-9, break down ECM components such as collagen and elastin, facilitating VSMC migration and proliferation, which are key contributors to neointimal hyperplasia [67–69]. Thrombin, in addition, as a central enzyme in the coagulation cascade, exacerbates thrombosis and vascular inflammation, further promoting ISR progression [70–72].

Enzyme activity in ISR is influenced by oxidative stress, pH changes and GSH levels. Elevated ROS levels in the early stages can excessively activate MMPs, worsening ECM degradation and vascular injury. As ROS levels decrease and the lesion stabilizes, MMP activity may subside, potentially reducing drug-release efficacy. Similarly, acidic conditions further enhance MMP activity, while elevated GSH levels in later stages of ISR can prematurely activate GSH-sensitive materials, disrupting controlled release [73, 74]. Targeting these enzymes offers a precise therapeutic approach to interrupt the ISR process at its most advanced stages.

#### Enzyme-responsive drug release

Enzyme-responsive drug delivery systems rely on the degradation of specific peptide bonds by targeted enzymes for sustainable or dynamic drug release (Figure 2).

##### Crosslinked peptide degradation

This degradation initiates the breakdown of multilayer bioactive molecule coatings [75], multi-phase nanocoatings [76], cerium dioxide (CeO_2_) hydrogel coatings [77], biodegradable stent surface coating based on poly-L-lactic acid [78], ZNF580 gene delivery systems [79–81] and NO donor nanofibers [82, 83]. As a result, bioactive agents like VEGF and encapsulated heparin are released, promoting endothelialization and providing antithrombotic effects, under MMP-2 enzyme-triggered conditions, the VEGF release rates were 17%, 32%, 51% and 70% on days 1, 3, 5 and 7, respectively, with a release duration of 12 days. Heparin release remained stable over time, with a total release period of approximately 50 days [75]. Additionally, the release of other bioactive factors, such as heparin and polylysine [76], facilitates locally prevent coagulation, enhances EC adhesion and stimulates cell proliferation and repair. CeO_2_ scavenges ROS [77], thereby reducing inflammation. In a 2 U/ml collagenase solution, CeO_2_ nanoparticles released 90% over 21 days, with rapid release in the first 2 days followed by slower release. After subcutaneous implantation in mice, the release reached 60% in 30 days. In a rabbit iliac artery/abdominal aorta implantation experiment, EC coverage in the poly(Sulfobetaine Methacrylate/Cerium Oxide Nanoparticles@Gelatin Methacrylate Dopamine Conjugate) (SBMA/CeO_2_@GMDA) group was 71.5% after 1 month, compared to 45.3% in the Poly-L-lactic acid (PLLA) group [77]. After 3 months, the poly(SBMA/CeO_2_@GMDA) group showed nearly 100% endothelial coverage, aligned with blood flow. ZNF580 promotes endothelialization, and NO inhibits platelet aggregation and neointimal hyperplasia [79–83]. Peptide amphiphile (PA)-YIGSR/KKKKK (YK)-NO nanofiber scaffolds increased EC proliferation from 51% to 67%, while smooth muscle cell proliferation decreased from 35% to 16%, which is critical for preventing ISR [82].

The peptide bonds in these coatings are cleaved by MMPs, resulting in the release of drug. Particularly, a polyelectrolyte multilayer membrane made from poly-L-lysine and methacrylated hyaluronic acid via layer-by-layer assembly is crosslinked with MMP-sensitive peptides (MMP-2 and MMP-9). This crosslinking allows dynamic mechanical adaptation, with MMPs softening the material to promote EC proliferation and monolayer formation. The initial hardness aids cell adhesion, while MMP-induced softening mimics natural ECM behavior, enhancing antithrombotic, anticoagulant and endothelial functions [84].

##### Thrombin cleavage

After stent implantation, thrombin binds to Arg-Gly peptide sequences in the crosslinked hydrogels, initiating their degradation and the controlled release of therapeutic agents. For example, thrombin-sensitive hydrogels release tissue-type plasminogen activator (t-PA) for fibrinolysis or heparin for anticoagulation [85–88]. In serum without thrombin, the release of t-PA was minimal, but with thrombin, the release rate increased significantly, showing a linear relationship to thrombin concentration. In low crosslinked hydrogels (DCL), under 10 U/ml thrombin conditions, the t-PA release rate was 0.077 mg/cm^2^·h, while in high DCL hydrogels, it was lower at 0.032 mg/cm^2^·h [85]. Similarly, thrombin-responsive nanogels degrade to release drugs like rivaroxaban [89], which inhibits clot formation, while Tempol and EGCG reduce oxidative stress and inflammation, promoting endothelialization and reducing ISR risk.

#### Practical challenges

The timing of enzyme activity is critical for effective drug release. In the early stages of ISR, thrombin levels drive coagulation and inflammation, necessitating the early release of anticoagulants. As the lesion progresses and MMPs become more active, they facilitate ECM breakdown and smooth muscle cell proliferation, which requires later-stage release of agents to stabilize the ECM and promote endothelial growth. To address these needs, thrombin-sensitive hydrogels can release anticoagulants early to prevent clot formation [85], while MMP-sensitive coatings can later release agents to stabilize ECM and promote endothelial growth [75]. Incorporating real-time enzyme monitoring could further refine release timing, enhancing therapeutic efficacy throughout ISR progression.

### Solvent-responsive materials

**Figure 3.**
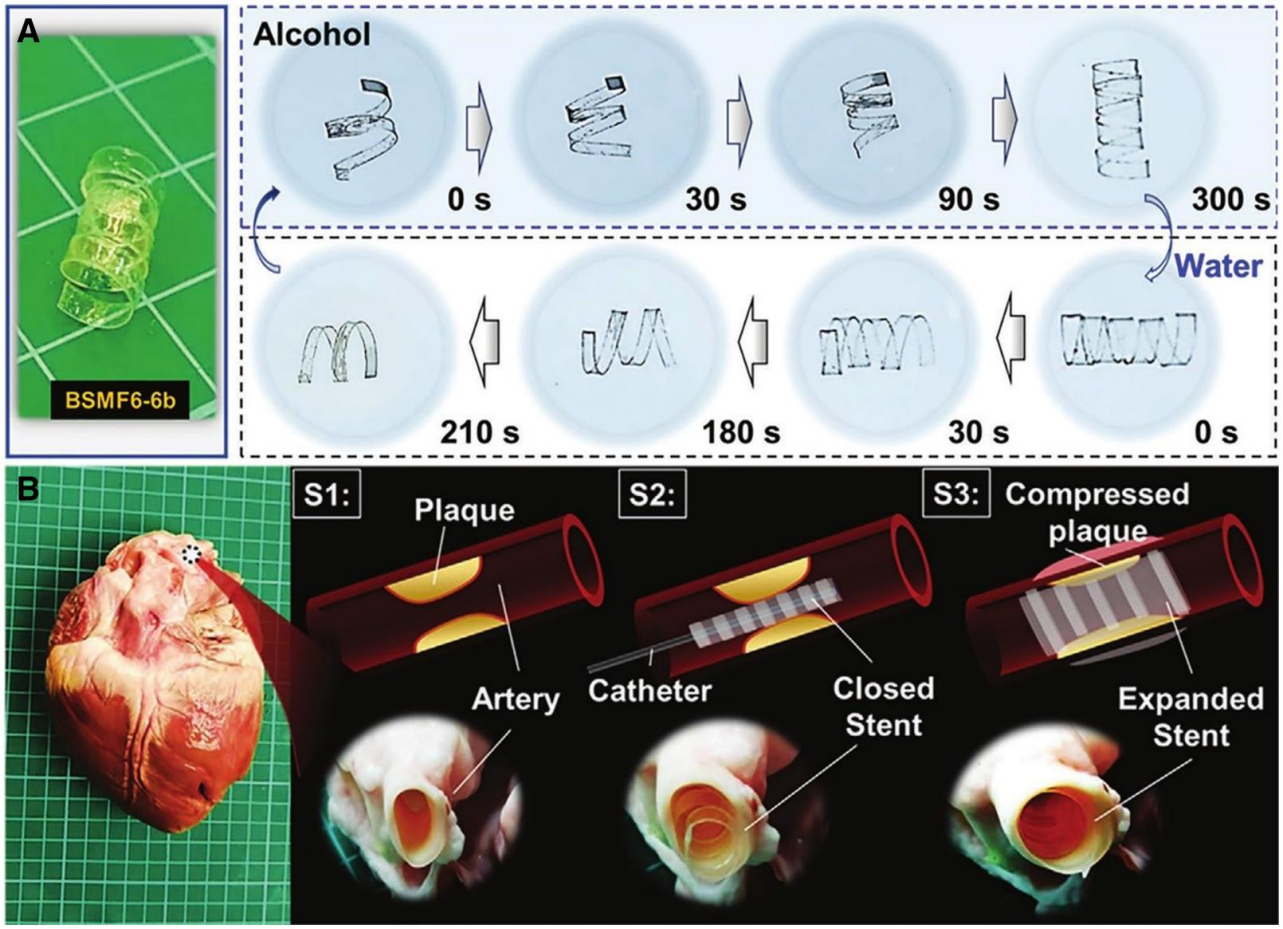
Solvent-responsive behavior of BSMF6-6b stent in alcohol and water. (**A**) Reversible coiling and expansion of the BSMF6-6b stent, coiling in alcohol within 300 seconds and expanding in water within 210 seconds. (**B**) *Ex vivo* application in a porcine heart model: S1 shows plaque in the artery; S2 shows the closed stent delivered via catheter; S3 shows stent expansion in water, compressing the plaque and restoring vessel patency. Adapted with permission from Shi et al. [93].

Solvent-responsive materials are a class of smart materials that alter their structure and properties in response to solvent exposure, making them valuable for biomedical applications like vascular stents and drug delivery systems. Zein, a hydrophobic plant-derived protein, degrades in a controlled manner in water or ethanol, with solvent infiltration occurring layer-by-layer, enabling sustained drug release without dependence on external concentration changes [90]. Hydration-responsive shape memory polymers (SMPs), enhanced with dopamine acrylamide (DAc), can soften and gradually conform to biological tissues under hydration, reducing mechanical mismatch and inflammation in implants [91]. The cell viability of 40 mol% DAc-modified polymers was 91.5% ± 10.8%. Under physiological conditions (37°C, PBS solution), the shape recovery rate (Rr) of 40 mol% DAc-modified SMP reached 90% within 20 seconds, whereas the unmodified SMP reached only 70% after 300 seconds [91]. Cellulose-based SMPs introduce a bidirectional, solvent-triggered shape change that allows reversible transformations in response to water and ethanol, providing repeatable adjustments that could be beneficial for stents needing multiple adaptations over time. Poly(vinyl alcohol) (PVA)-based SMPs, with their programmable, multi-step shape recovery controlled by surface wettability, offer staged expansion, ideal for applications like phased drug release [92, 93]. Bilayer two-way shape memory films (BSMFs) underwent compression load testing, showing a maximum load of 3.6 N, meeting the requirements for use as an artificial vascular stent. BSMFs demonstrated rapid shape adjustment with response times of 210 seconds in water and 300 seconds in ethanol [93] (Figure 3). Biodegradable poly(fumaric acid) copolymers (PBF) and water-responsive PVA derivatives add the advantage of predictable degradation, making them suitable for temporary implants. PBF’s functional groups enable growth factor loading and controlled release, supporting tissue engineering [94].

### Practical challenges

A common limitation to these materials is the one-off shape recovery, typically triggered by initial exposure to the solvent [91–94]. It restricts their effectiveness in chronic, progressive conditions like ISR, where continuous adaptation may be needed as the vascular environment evolves. Some materials degrade too quickly. For example, PBF material loses 62.7% of its mass within 7 days in enzyme solution. Rapid degradation may lead to premature failure of the stent structure [94]. Additionally, most solvent-responsive materials lack multi-responsiveness, responding only to hydration or a specific solvent, rather than adapting to diverse physiological signals such as pH, enzyme activity, or oxidative stress. To overcome these limitations, composite designs that incorporate multi-responsive elements (e.g. enzyme- or pH-sensitive domains) could enable materials to adjust shape or release drugs over time in response to local conditions. Alternatively, surface modifications to control solvent absorption could enable a staged response, extending the material’s support in dynamic environments like ISR. By integrating these strategies, solvent-responsive materials could be further developed to offer multi-step recovery, bidirectional adaptability, and tunable degradation, providing more durable, adaptable stents and drug delivery systems [90–94].

## Smart stent materials responding to external stimuli

### Light

**Figure 4.**
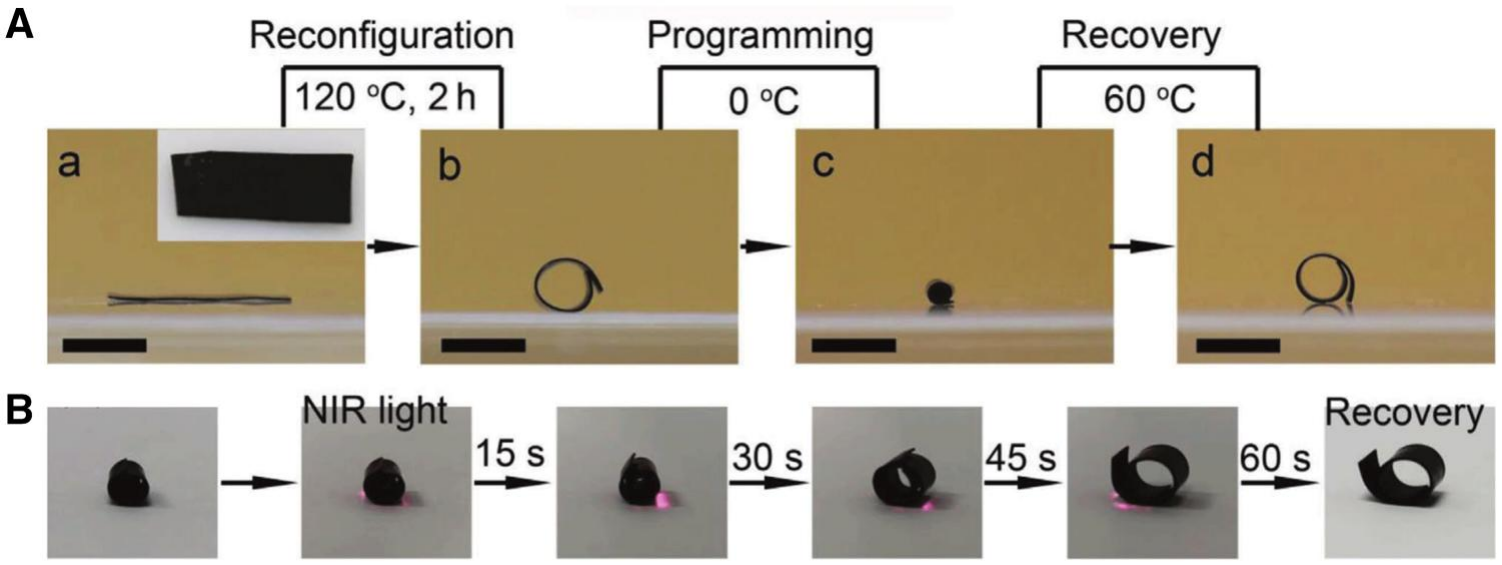
NIR-triggered shape memory effect in SMP-based vascular stents. (**A**) Stepwise thermal programming and recovery of SMP showing reconfiguration at 120°C, shape programming at 0°C, and shape recovery at 60°C. (**B**) Time-sequence images of SMP under NIR light (808 nm) showing shape recovery from a programmed state to its original shape within 60 seconds, demonstrating the material’s responsiveness and potential for repeatable stent expansion in vascular applications. Adapted with permission from Liang et al. [95].

Light-responsive materials are in an exploratory phase for vascular stent applications, particularly through near-infrared (NIR) light activation, which holds the potential for non-invasive, repeatable stent expansion. Under 808 nm laser irradiation, the material temperature increased from 35.7 to 94.7°C, with a faster temperature rise as laser power density increased. At a laser power of 0.52 W/cm^2^, the material temperature reached over 60°C in just 29 seconds, sufficient to trigger shape recovery [95] (Figure 4). Upon NIR irradiation, these materials can restore their initial expanded shape [96]. This repeatable expansion capacity offers a promising solution for non-invasive ISR treatment, allowing stents to be re-expanded to restore vessel patency without additional surgery. Additionally, these materials show adaptability for complex vascular anatomy; for instance, stents can be initially designed in a flexible, compact form to navigate tortuous or narrow vessels and then expanded to a stable supporting structure upon NIR activation, ideal for deployment in coronary or cerebral arteries where conventional rigid stents face limitations [96].

### Practical challenges

Despite the promising potential, a key challenge for NIR-activated stents is the high activation temperature, often around or above 55°C, which exceeds safe thresholds for vascular tissues and risks thermal damage [95–98]. Reducing activation temperature through material optimization remains crucial for clinical feasibility. However, there is currently a gap in the exploration of temperature-sensitive materials specifically designed for vascular stents. The lack of materials that can effectively lower activation temperatures while maintaining the stent’s mechanical properties represents a significant research opportunity.

Additionally, the limited tissue penetration of NIR light, along with shielding by structures like the skull and sternum, restricts its application to peripheral or superficial arteries, making it less suitable for deeper, critical vessels like coronary or cerebral arteries [95–99]. To address these challenges, possible activation methods include percutaneous irradiation for superficial applications, fiber-optic NIR conduits for deeper vessels requiring minimal invasion, or implantable micro-NIR devices that can activate the stent *in situ*. The optimization of NIR light absorption efficiency and the development of advanced materials that can activate at lower temperatures are essential steps toward improving the clinical applicability of NIR-responsive stents.

### Magnetic

In recent years, to improve the success rate of stent implantation in distal and complex blood vessels, researchers have advanced magnetically controlled wireless materials, reducing reliance on conventional wires and catheters [100]. By combining Fe_3_O_4_ nanoparticles with shape memory materials, these systems leverage the magnetocaloric effect to achieve precise expansion and contraction of stents, facilitating navigation through narrow, tortuous vessels with greater intraoperative stability. This magnetocaloric approach has also advanced drug delivery systems, enabling controlled release by altering the polymer structure under a magnetic field.

**Figure 5.**
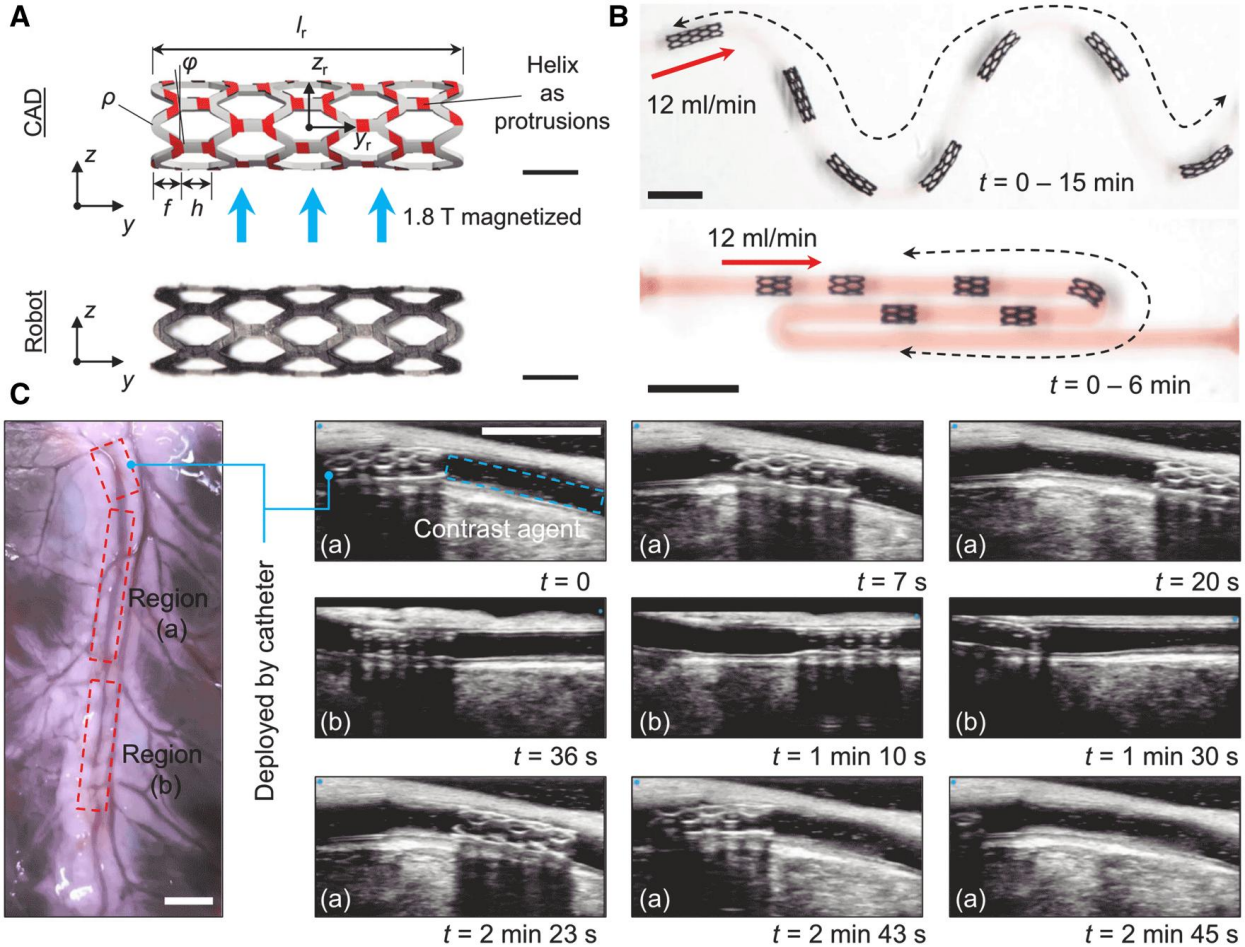
Magnetically controlled soft robot design and performance in complex vascular pathways. (**A**) CAD model and prototype of a magnetically actuated soft robot with a stent-like structure, designed for precise navigation and controlled radial expansion under an external magnetic field (1.8 T). (**B**) Demonstration of robot navigation through a tortuous vascular model with a flow rate of 12 ml/min, illustrating adaptability to complex paths. (**C**) Real-time ultrasound imaging of the robot in an *ex vivo* porcine vascular model, showing deployment and tracking over time with contrast agent-enhanced visibility. Adapted with permission from Wang et al. [102].

Recent developments in magnetically responsive shape memory materials, including SMPs integrated with ferromagnetic nanoparticles, support precise remote control for vascular applications [100–102] (Figure 5). Systems fabricated with 4D printing techniques are designed for wireless operation, allowing controlled actuation and phase-change responses that trigger radial expansion upon heating. Fe_3_O_4_ nanoparticles embedded in SMPs facilitate localized heating under magnetic field exposure, initiating shape memory activation at temperatures between 38 and 50°C and producing mechanical outputs of up to 70 N with a power density of 175.2 J·g^(−1)^ [100, 101]. This functionality enables controlled navigation through complex vascular pathways. The materials’ bi-stable design supports repeatable expansion and contraction cycles, preventing failure or fatigue and greatly enhancing the material's durability and long-term performance. In addition, it can change shape and move within the blood vessel, reducing the dependence on catheter-based delivery techniques [100].

Magnetothermally responsive systems, such as superparamagnetic iron oxide nanoparticles, are utilized for targeted delivery under alternating magnetic fields, improving therapeutic outcomes, as shown in recent studies on magnetic nanosystems [103]. The magnetic field induces heating above the lower critical solution temperature (LCST) of the polymer, approximately 40.1°C, triggering a hydrophilic conversion that initiates drug release and enhances therapeutic efficacy. At 43°C, the cumulative drug release reaches 86% within 4 hours and 97% within 12 hours. In contrast, at physiological temperature (37°C), the release is slower, with only about 35% cumulative release over 8 hours. The nanocarriers’ magnetothermal properties are optimized to rapidly heat to 42.9°C under an alternating magnetic field, significantly above physiological temperature. The LCST is precisely controlled at 40.1°C by adjusting the N-Isopropyl acrylamide (NIPAAm) to N-hydroxymethyl acrylamide (HMAAm) ratio, enabling efficient drug release near body temperature [104]. A novel approach combines FeRh alloy with the thermosensitive polymer poly-N-isopropylacrylamide (PNIPAm) in implant coatings. Exposure to a 1–3 T magnetic field decreases the coating temperature by 2–7°C, facilitating a transition from hydrophobic to hydrophilic, triggering drug release [105]. Additionally, the integration of magnetic nanoparticles with thermosensitive polymers enables more precise drug delivery by utilizing the magnetocaloric effect [106, 107]. Low-intensity magnetic fields can induce localized temperature changes, facilitating the release of drugs in targeted areas and overcoming biological barriers like cell membranes [107]. Furthermore, hotspot effects generated by the magnetic field allow for even more localized control, improving the efficiency and precision of drug release [108].

### Practical challenges

Magneto-responsive systems face several critical challenges in clinical applications, particularly with respect to scalability, manufacturability, and energy requirements. One key challenge is the balance of radial force with flexibility while also maintaining biocompatibility and ensuring stability in vascular pathways [101–103]. To address this, magneto-/electro-responsive polymers (MERPs) and magnetoactive soft materials (MSMs) offer a potential solution, as they can be engineered to adapt dynamically to varying forces while maintaining the necessary mechanical properties for flexibility and stability within biological environments [109, 110]. In addition to the magnetic field, MSMs can also combine with other stimuli, such as humidity, light and heat, to achieve more complex driving behaviors.

Further challenges in magnetocaloric drug delivery include the need for precise temperature control and efficient management of tissue heat dissipation [104, 106, 108]. MERPs and MSMs, with their ability to respond to external magnetic fields, can address these challenges by controlling the heat generation during the drug-release process [109, 110]. However, high magnetic field requirements introduce technical and safety challenges in clinical settings. These systems also face potential issues with medical imaging interference, especially in magnetic resonance imaging, due to the magnetic properties of the materials.

To overcome these issues, future research should focus on refining magnetic field parameters [107], improving surface smoothness to reduce vascular injury and developing temperature-responsive materials that are both reliable and clinically safe. Additionally, advancements in 3D/4D printing could further enhance the scalability and manufacturability of MERPs and MSMs, making them viable for widespread clinical applications [109, 110].

### Ultrasound

**Figure 6.**
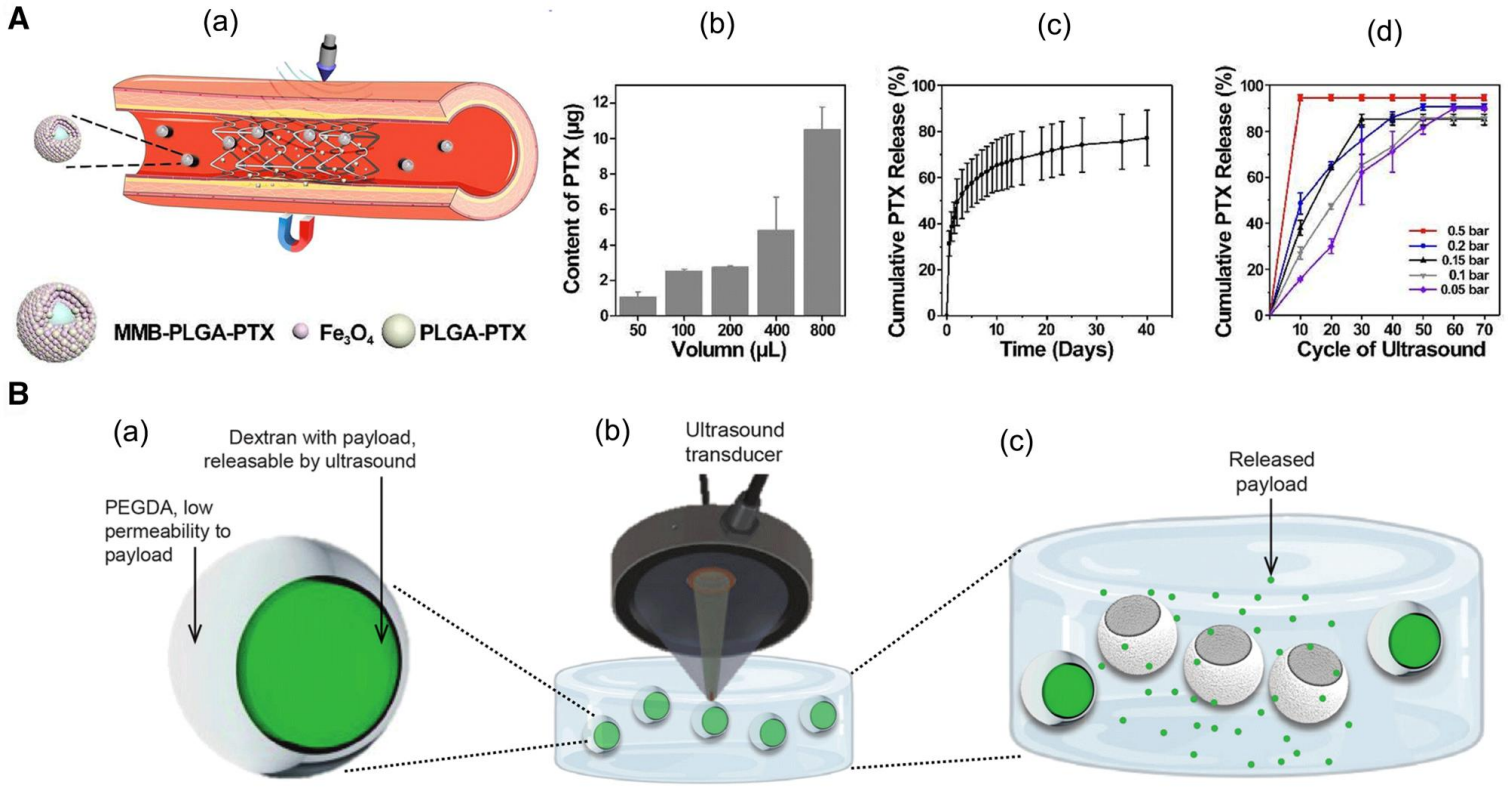
Ultrasound-responsive microcapsules for targeted drug release. (**Aa**) Schematic of microbubble-assisted drug delivery system, illustrating a vascular stent with microbubble-encapsulated PLGA-paclitaxel (PLGA-PTX) particles, guided to the target area using magnetic nanoparticles (Fe_3_O_4_) and activated by ultrasound. (**Ab**) Quantification of PTX content loaded in microbubbles at different preparation volumes, demonstrating scalable drug loading. (**Ac**) Cumulative PTX release profile over time, showing sustained drug release. (**Ad**) Cumulative PTX release under various ultrasound cycles and pressures, indicating controlled release with increased cycles and pressure. Adapted with permission from Wang et al. [113] (**Ba**) Cross-sectional view of ultrasound-responsive microcapsule with a dextran core containing the drug payload, surrounded by a PEGDA shell, designed to restrict passive drug release. (**Bb**) Illustration of focused ultrasound (FUS) activation, where the ultrasound transducer selectively targets microcapsules, triggering cavitation within the dextran core. (**Bc**) Release of drug payload post-ultrasound activation, illustrating the mechanism for on-demand drug delivery. Adapted with permission from Field et al. [111].

Recent advancements in drug delivery technology have explored ultrasound-responsive microcapsules to achieve precise, controlled release of therapeutic agents. These systems rely on encapsulating drugs within polymeric microcapsules that respond to focused ultrasound to trigger drug release through structural changes driven by inertial cavitation and mechanical shear forces. Gold nanoparticle-enhanced microcapsules respond to ultrasound through inertial cavitation, where gas bubbles collapse, generating shock waves and shear stress that rupture the capsules, releasing drugs precisely when needed. Another system employs biphasic hydrogel microcapsules with a dextran core and a poly(ethylene glycol) diacrylate (PEGDA) shell, where ultrasound triggers stepwise, controlled drug release [111, 112]. In a further advancement, a magnetically guided system was developed for treating ISR. This method combines magnetic nanoparticles and poly(lactic-co-glycolicacid) (PLGA) nanoparticles loaded with PTX. Magnetic fields guide the microbubbles to the stented area, and low-intensity focused ultrasound (LIFU) induces microbubble oscillations, releasing the drug. Under physiological conditions (0.1M PBS, pH 7.4, 25°C), PLGA-PTX nanoparticles exhibited an initial rapid release of approximately 50%, followed by a sustained release, reaching about 70% over 15 days. Under 0.5 bar acoustic pressure, after 10 ultrasound cycles, the PTX release reached 90%, whereas under 0.05 bar acoustic pressure, the release was only 16% [113] (Figure 6). These oscillations, combined with microstreaming and mechanical forces, enhance deep drug penetration into the arterial wall and extend the drug’s retention time, overcoming the issue of rapid clearance by blood flow [111–113]. In addition, dual-responsive systems incorporating both pH and ultrasound sensitivity in polymeric micelles and magnetic nanoparticles further enhance drug-release precision. These systems offer stepwise drug release and improved tissue penetration, making them highly effective for targeted delivery in tumor treatment and vascular interventions [114, 115].

### Practical challenges

In ultrasound-mediated drug delivery, routine applications like echocardiography and carotid ultrasound typically use low ultrasound intensities (0.1–0.5 W/cm^2^), which are safe for imaging without affecting blood flow or vascular walls [116]. In contrast, LIFU used for drug delivery operates at higher intensities (1–3 W/cm^2^), triggering microbubble cavitation and enhancing drug release. While effective for targeted therapy, higher intensities can pose risks, including blood clot formation and vascular damage, particularly in deep vessels such as the coronary or cerebral arteries. Therefore, increasing ultrasound intensity requires careful control and preclinical validation to ensure safety and avoid adverse effects like thrombus formation.

### Temperature

Temperature-responsive materials are increasingly explored for vascular stent applications, using temperature as an exogenous trigger (e.g. radiofrequency (RF)-induced or magnetocaloric heating) or as an endogenous response within physiological limits [117, 118]. These materials harness temperature shifts to activate shape memory properties, facilitate controlled drug release, or adjust mechanical compliance to mitigate ISR. For example, RF-controlled stents can achieve localized heating through a resonant circuit to enable precise, on-demand drug release, offering more controlled activation compared to ambient body temperature [118]. Another approach leverages magnetocaloric effects to achieve targeted drug release within specific vascular areas, providing a localized therapeutic strategy under external magnetic field activation [117].

Certain materials, particularly self-healing polymers and multi-segmented structures, offer extended functionality and durability, addressing the challenges of maintaining vascular implant stability over time. For instance, a self-healing polymer can autonomously repair micro-damage, preserving structural integrity under dynamic vascular conditions [119]. Multi-segmented constructs, such as 4D-printed β-CD-grafted PCL stents, demonstrate enhanced drug-loading and sustained release properties, extending therapeutic efficacy *in situ*. Each stent was loaded with 80 µg of PTX. The release showed an initial burst of 28.59% in the first 3 days, followed by a gradual decrease, reaching 53.81% over the next 27 days, lasting over a month. This prolonged release period is longer than traditional DES, such as the Taxus stent, which releases the drug within 15–20 days. In *in vitro* experiments, the material’s degradation rate was 3.5% within 1 month, with minimal impact on material properties or solution pH [120].

### Practical challenges

However, the application of temperature-responsive stents in vascular interventions is constrained by the need to maintain activation temperatures at or below 40–43°C to avoid endothelial damage or thrombogenesis in sensitive vascular tissues. The body’s stable internal temperature (37°C) limits these materials to single-use activation upon implantation rather than allowing continuous responsiveness [117–120]. The practical feasibility of these materials is currently more favorable in peripheral vascular applications, where localized external heating can be applied with minimal impact on critical tissues. For example, peripheral vessels, such as those in limbs, are more accessible to non-invasive heat application methods (e.g. topical heating), whereas in critical vessels like cerebral or coronary arteries, safety concerns and the risk of collateral tissue damage make implementation more challenging. In the future, consideration can be given to adjusting the molecular weight of PCL or introducing other polymers with stronger temperature responsiveness to lower the activation temperature of the material, bringing it closer to human body temperature.

### Electricity

**Figure 7.**
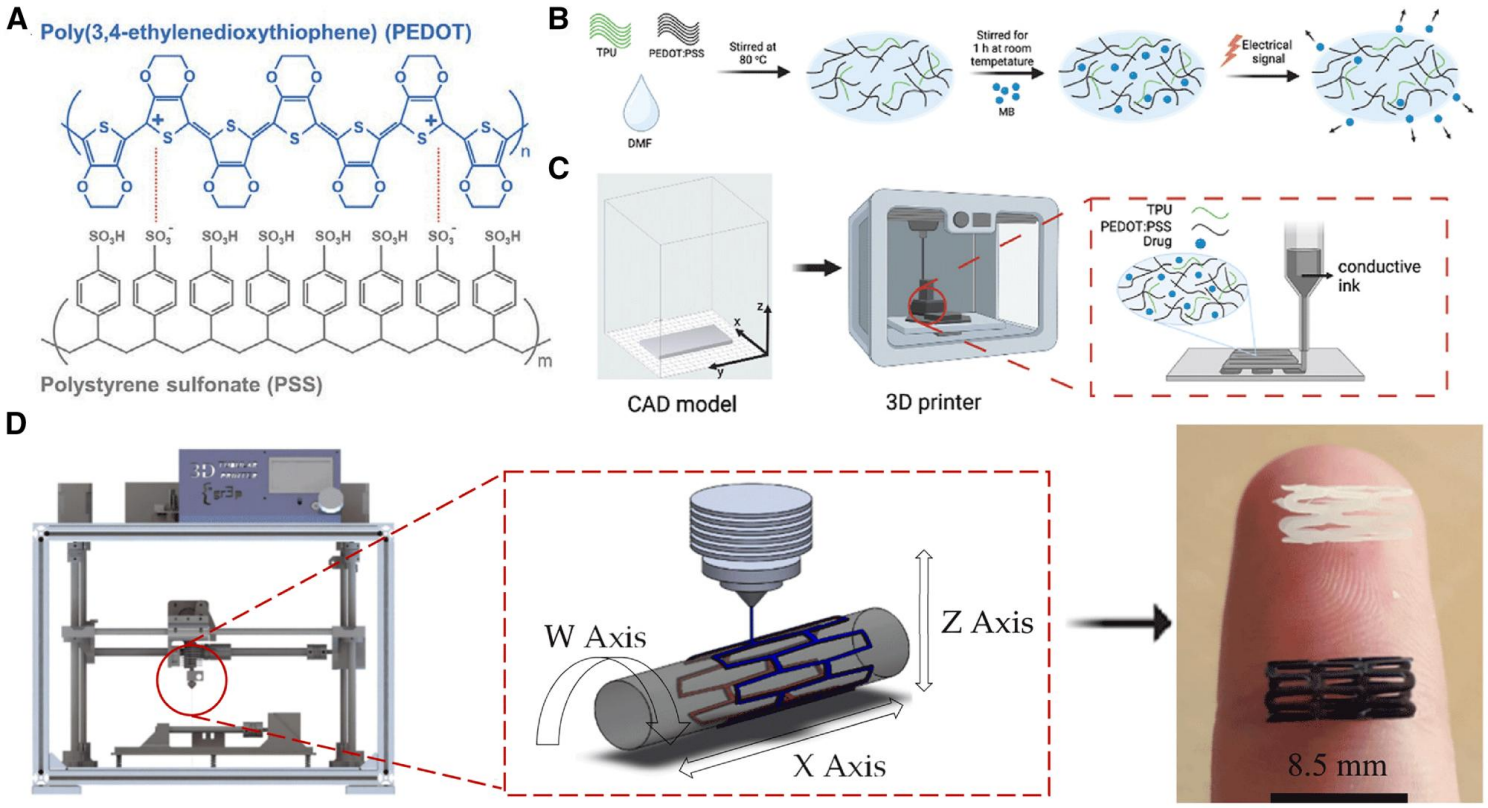
Development and manufacturing of electro-responsive 3D-printed stents using conductive polymer composites. (**A**) Chemical structure of PEDOT:PSS for enhanced solubility and conductivity. (**B**) Preparation of PEDOT:PSS/TPU composite with embedded drug molecules for controlled release. (**C**) CAD modeling and 3D printing process for creating stents. (**D**) Schematic of the 3D tubular printer mechanism employing PCL/PLA composite materials. Adapted with permission from Alkahtani et al. [121] and Guerra et al. [126].

Recent studies have demonstrated the potential of combining the conductive polymer poly(3,4-ethylenedioxythiophene) (PEDOT) with various biocompatible matrices, such as thermoplastic polyurethane, alginate and PCL, to create flexible, electrically responsive drug delivery systems. Using PEDOT, these systems leverage the polymer’s electroactivity to enable responsive drug release, tailored by applying low voltage (±0.5 to ±1.0 V) to release drugs in pulsatile or continuous patterns as needed [121–126] (Figure 7). PEDOT’s versatility allows it to be integrated as conductive nanoparticles in different material forms, including hydrogels, electrospun fibers and 3D-printed matrices. For instance, PEDOT nanoparticles embedded in hydrogels or nanofibers act as ‘microactuators’, expanding or contracting under electric stimuli to release hydrophobic drugs like curcumin and chloramphenicol in a controlled manner [122–126]. This approach enables PEDOT-based systems to adjust drug delivery rates by modifying voltage, offering a significant advantage in vascular treatments where controlled, on-demand release is essential. Moreover, studies have shown that PEDOT’s conductive properties remain stable in physiologically relevant environments, enhancing its potential for long-term use in implantable devices [121, 122]. Moreover, some other conductive polymers, such as polyaniline and aniline trimer, exhibit oxidation reactions when a reduced voltage is applied to the hydrogels they form. This process causes the hydrogel's network structure to expand or loosen, providing pathways for drug release [125, 127–129]. For example, under 3 V electrical stimulation, the cumulative release of dexamethasone reached 90% within 120 minutes (Dextran-Aniline Trimer 10% - Hexamethylene Diisocyanate) (Dex-AT10/HDI), significantly higher than 35% under no electrical stimulation. The cumulative release of indomethacin also reached 27% within 120 minutes (Dex-AT10/HDI), significantly higher than 9% under no electrical stimulation [129]. Additionally, graphene-incorporated hydrogels exhibit pulsatile drug release through the reversible swelling and shrinking behavior of their matrix when an electric field is applied. Moreover, graphene demonstrates remarkable heat dissipation capabilities, which, compared to other systems, prevent harmful resistive heating and reduce the risk of potential tissue necrosis [130].

### Practical challenges

While these low-voltage requirements make PEDOT systems promising for peripheral vasculature applications, challenges remain in extending their use to sensitive areas such as cardiac and cerebral vessels, where electric fields could interfere with electrophysiological activity, potentially leading to arrhythmias or seizures. To address this, future research must focus on optimizing insulation, achieving precise current localization and limiting applications to non-critical vasculature until safety in sensitive tissues is validated. Further advancements in integrating PEDOT with more biocompatible, electrically insulating materials could improve safety and broaden the scope of these innovative electricity-responsive drug delivery systems. During electrical stimulation, the material may experience ‘resistive heating’, leading to an increase in temperature, which could potentially cause damage to surrounding tissues. Future research is needed to explore novel thermally conductive materials or composites to improve thermal conductivity and reduce damage.

## Summary and perspectives

Recent strategies for addressing ISR focus on developing stimuli-responsive materials, which are expected to adapt to the biological environments associated with ISR [17]. By responding to endogenous and exogenous triggers, smart materials aim to enhance therapeutic efficacy and improve biocompatibility, paving the way for future clinical applications [18] as listed in Table 2.

**Table 2.** Stimuli and their roles in addressing ISR mechanisms

Trigger	Targeted ISR processes	Responsive mechanisms of material	Effectiveness and challenges
pH (acidic environments)	VSMC activation, ECM remodeling, foam cell formation	Acid-sensitive bond cleavage enables localized drug release and anti-inflammatory effects	Effective for localized delivery, but limited by material stability; challenges in maintaining controlled release across dynamic physiological pH gradients
Redox imbalance (ROS, GSH)	ECM degradation, foam cell formation	ROS-mediated bond cleavage for ECM disruption; GSH-triggered release of healing agents	Highly effective in oxidative stress conditions but requires precise control over ROS and GSH levels. Risks of oxidative tissue damage and inflammatory responses from degradation byproducts
Enzyme activity (MMPs, thrombin)	ECM degradation, VSMC activation	Enzymatic degradation of ECM components, enhancing localized drug action	Targeted and selective, but limited reliability due to interpatient enzyme variability; unresolved biocompatibility concerns
Solvent exposure	ECM structural alteration, VSMC activation	Structural reconfiguration and drug release in response to solvent exposure	Useful for dynamic control, but practical application is hindered by the need for precise solvent delivery and potential tissue irritation
Light (NIR)	Stent expansion, structural recovery	NIR-activated shape recovery allows non-invasive stent expansion	Non-invasive and precise, but limited by poor tissue light penetration and safety risks from prolonged irradiation
Magnetic fields	Targeted navigation, stent positioning	Magnetic field-induced movement of the stent, allowing targeted positioning and controlled drug release	Practical for non-invasive positioning, but requires high-strength magnetic fields and specialized equipment; interference from tissue heterogeneity
Ultrasound	Control of VSMC proliferation, foam cell formation	Ultrasound-induced cavitation enabling localized drug delivery	Highly effective for controlled release, but requires precise control over ultrasound parameters to avoid tissue damage
Temperature	ECM remodeling, VSMC activation	Temperature-responsive activation of shape memory properties facilitating expansion of the stent	Reliable in controlled settings, the fluctuation in temperature may lead to systemic adverse effects, limiting its widespread application
Electricity	ECM remodeling, endothelial proliferation, VSMC migration	Low-voltage stimulation inducing specific changes in cellular behavior by promoting localized drug effects	Effective for tissue-specific responses, but long-term safety concerns and inconsistent response in chronic applications

By utilizing stimuli-responsive materials, these intelligent stents can detect changes in the *in vivo* environment and respond by releasing drugs or adaptively adjusting their structure, thereby more effectively inhibiting ISR-related processes like smooth muscle cell proliferation and thrombosis. While these innovative materials have shown promising potential, their journey from preliminary studies to clinical application is complex, with many still undergoing basic testing or existing only as early-stage prototypes of stents as listed in Supplementary Table S1. Bridging these gaps will require addressing specific challenges across development stages.

At the initial material testing phase, many studies focus on fundamental properties such as biocompatibility, mechanical stability, drug-release controllability and toxicity and have yet to reach the stage of stent prototyping. Some materials were originally developed for other diseases (e.g. cancer, inflammation), but their drug-release mechanisms show promise for ISR pathology, making them intriguing concepts with potential applications. At the stent prototype stage, some materials have successfully been shaped into stents, but there are significant differences in their level of completion across *in vitro*, animal and human studies. For materials that have been fully developed into stent structures, challenges remain in achieving precise drug release, controlled degradation rates, and compatibility with complex vascular environments.

Future development should focus on improving the biocompatibility and degradation rates of these smart materials, optimizing precise control over drug release, and advancing their validation through cell, animal and human studies. Additionally, the application of advanced manufacturing and testing technologies, such as 3D printing and microfluidic chip technology, holds the potential to accelerate the development and evaluation processes. Moreover, the journey from early-stage prototypes to clinical use involves overcoming substantial regulatory challenges. The dual nature of these materials as both medical devices and drug delivery systems adds complexity to their approval process, requiring compliance with both device and pharmaceutical regulations. In addition, ensuring the long-term safety, stability and biocompatibility of these materials, alongside precise control over their drug release and degradation rates, will be critical for their successful market adoption. Meeting these regulatory requirements will demand rigorous testing and validation at every stage, as well as the development of innovative testing alternatives such as organ-on-a-chip systems to better simulate human physiological conditions, reduce animal testing and expedite regulatory review processes.

Finally, although most smart stent technologies do not currently integrate sensors, there is future potential in incorporating micro-sensors to monitor physiological states around the stent in real time, enabling more precise drug release or therapeutic adjustments when necessary. Though still in the research and early testing phase, this advancement could eventually open new avenues in-stent-based treatments. Widespread clinical application will also require overcoming challenges related to production scalability, cost control and market acceptance. Through promoting interdisciplinary collaboration and international exchange, smart stent technology holds the potential to further enhance the efficacy of stent therapy in the future, offering a precise and sustainable solution to reduce ISR incidence.

## Supplementary Material

rbaf020_Supplementary_Data

## Data Availability

No data was used for the research described in the article.
