# Supplementary material for: Smart materials strategy for vascular challenges targeting in-stent restenosis: a critical review
Source: Regen Biomater. 2025 Mar 24;12:rbaf020. doi: 10.1093/rb/rbaf020 (PMC12034381; doi:10.1093/rb/rbaf020)
Supplement: rbaf020_Supplementary_Data [file rbaf020_supplementary_data.zip › Supplementary_Table-e1.docx]

Supplementary Data:

**Smart Materials Strategy for Vascular Challenges Targeting In-Stent Restenosis: A Critical Review**

Authors: Kai Zhang^1^, Wenzhao Liang^1^, Xiao-Bo Chen, Jing Mang,*

**Table e1 Key studies on responsive materials for ISR and their development stage**

| **Study** | **Material** | **Material Type** | **Application** | **Development Stage** | **Targeted ISR Mechanism** | **Key Findings/Progress** | **Primary Challenges** |
| --- | --- | --- | --- | --- | --- | --- | --- |
| Wang K, 2021 | ROS-responsive coating | EGCG/ Cysteine/Hydrochloride/ Pitavastatin Calcium | Dynamic drug release for ISR | ECs and SMCs testinging/Animal Model with a stent prototype | VSMC proliferation, Inflammation | Showed good biocompatibility and anti-stent restenosis ability in animal experiments | Requires selective VSMC inhibition while preserving endothelial function. |
| Han X, 2023 | ROS-responsive coating | HA/Cystamine/ Allicin | Controlled drug release for ISR | ECs and SMCs testing /Animal Model with a stent prototype | VSMC proliferation, Inflammation | Promoted endothelial repair and reduced neointimal formation. | The H_2_S released by allicin may be unstable and influenced by the environment |
| Yan H, 2024 | ROS-responsive coating | MA(PCLA)/PEI-TK-Dex/ PDA | Dynamic drug release for ISR | ECs and SMCs testing /Animal Model with a fully formed stent | VSMC proliferation, Inflammation, Thrombosis | Achieved ROS-triggered self-regulated drug release with anti-inflammatory effects. | Coating effects on vascular smooth muscle cell proliferation require optimization |
| Duan L, 2023 | ROS-responsive coating | Sodium Alginate/ DSPE-PEG/ Exosomes | Controlled drug release for ISR | ECs and SMCs testing /Animal Model with a stent prototype | VSMC proliferation, Inflammation, Thrombosis | Reduced inflammation and VSMC proliferation in animal and cellular models | Long-term biocompatibility and exosome collection efficiency need validation. |
| Ye W, 2019 | GSH-responsive coating | Sulfhydryl-Containing Polycation/ pVEGF | Targeted drug delivery for ISR | ECs testing/Animal Model with a fully formed stent | Thrombosis | Showed good blood compatibility, reduced occurrence of restenosis in animal models | Localized high ROS environment may influence nucleic acid release |
| Zhang B, 2023 | GSH-responsive coating | Zn²⁺/ Lysozyme | Continuous NO release for ISR | ECs and SMCs testing /Animal Model with a fully formed stent | VSMC proliferation, Inflammation, Thrombosis | Achieved sustained NO release and stability in animal models | Oxidative stress may interfere with NO release kinetics |
| Wang K, 2022 | Redox-responsive coating | EGCG/Cystamine | Controlled drug release for ISR | ECs and SMCs testing /Animal Model with a stent prototype | VSMC proliferation, Inflammation, Thrombosis | Enhanced endothelial proliferation and SMC inhibition | Response variability in complex pathological microenvironments |
| Li P, 2022 | Redox-responsive coating | Tellurium functionalized polycarbonate polyurethane | Continuous NO release for ISR | ECs and SMCs testing /Animal Model with a fully formed stent | VSMC proliferation, Inflammation, Thrombosis | Significant anticoagulation and anti-inflammatory effects in animal models | Long-term biocompatibility and coating stability in local microenvironments |
| Yang F, 2023 | Enzyme-responsive coating | CeO₂/GelMA/ SBMA | Controlled drug release for ISR | ECs testing/Animal Model with a fully formed stent | Oxidative stress, Inflammation, Thrombosis | Formed intact endothelial layer on stent surface in animal models | Coating degradation in long-term pathological environments |
| Zhao J, 2022 | Enzyme-responsive coating | pZNF580/ Dopamine /MCP/LMW-PEI/ F-ssPEI | Targeted gene delivery for ISR | ECs and SMCs testing /Animal Model with a vascular graft | Thrombosis | Accelerated vascular graft repair in animal models | Excessive oxidative stress impairs the transfection process |
| Zhou J, 2021 | Enzyme-responsive coating | pZNF580/ MCP /mPEG-b-PLMD-g-PEI-PEG-biotin | Targeted gene delivery for ISR | ECs testing/Animal Model with a vascular graft | Thrombosis | Achieved uniform neointima formation in animal models | Excessive oxidative stress impairs the transfection process |
| Maitz MF, 2024 | Enzyme-responsive coating | starPEG/heparin/thrombin-cleavable peptide | Controlled drug release for ISR | Animal Model with a fully formed stent | Thrombosis | Reduced thrombosis on neurovascular devices.in animal models | Blood compatibility requirements vary under different shear conditions |
| Wang Y,2022 | Enzyme-responsive coating | R@NG/EGCG/Tpl/ Rivaroxaban | Controlled drug release for ISR | ECs and SMCs testing /Animal Model with a fully formed stent | VSMC proliferation, Thrombosis, Inflammation, Oxidative stress | improved stent mechanical properties and reduced stenosis rate | Long-term biocompatibility |
| Liang R,2021 | Light-responsive polymers | Polyethylene glycol/ Dopamine/ Fe³⁺ | Mechanical force adjustments, shape memory | Mechanical testing with a stent prototype | Mechanical failure | Achieved precise shape recovery under NIR | Limited tissue penetration depth and risk of thermal damage |
| Chu C, 2020 | Light-responsive polymers | 6A PEG-PCL / YHD798 | Mechanical force adjustments, shape memory | Mechanical testing /Animal Model with a fully formed stent | Mechanical failure | Achieved precise shape recovery under NIR | Limited tissue penetration depth and risk of thermal damage |
| Zhou Y, 2021 | Temperature-responsive polymers | PCL/β-CD | Mechanical force adjustments, dynamic shape change | ECs testing/ physical property evaluation of a fully formed stent | Mechanical failure,  VSMC proliferation | High mechanical strength with controlled drug release | Paclitaxel (PTX) release may inhibit vascular endothelial cell proliferation |

Abbreviations: Epigallocatechin gallate :EGCG , HA :Hyaluronic Acid，MA(PCLA)：poly(2-methacryloyloxyethyl phosphorylcholine-lauryl methacrylate-methacrylic acid) amphoteric polymers, PEA：polyethyleneimine, TK：thioketal，Dex: dexamethasone, PDA: polydopamine, VEGF: vascular endothelial growth factor, GelMA: gelatin methacryloyl, SBMA: sulfobetaine methacrylate, MCP：MMP-cleavable peptide, F-ssPEI：fluorinated cationic polymer，starPEG: four-armed poly(ethylene glycol), R@NG: nanogels prepared by cross-linking between the amino group of cleavable peptide [NH2-Gly-(D)CHA-Ala-Arg-Ser-TrpGly-CONH2] and the aldehyde group of Ox-HA, 6A PEG-PCL: 6-arm poly(ethylene glycol)–poly(e-caprolactone), PCL: polycaprolactone, β-CD: β-cyclodextrin, ECs: endothelial cells, SMs: smooth muscle cells, VSMC: vascular smooth muscle cell, ISR: in-stent restenosis.
