# Supplementary material for: Smart materials strategy for vascular challenges targeting in-stent restenosis: a critical review
Source: Regen Biomater. 2025 Mar 24;12:rbaf020. doi: 10.1093/rb/rbaf020 (PMC12034381; doi:10.1093/rb/rbaf020)
Supplement: rbaf020_Supplementary_Data [file rbaf020_supplementary_data.zip › Supplementary Files.pdf]

RightsLink

ZK

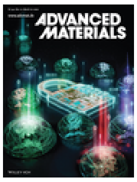

An Innovative Solvent-Responsive Coiling–Expanding Stent

Author: Shuo Shi, Miao Cui, Fengxin Sun, et al

Publication: Advanced Materials

Publisher: John Wiley and Sons

Date: Jul 4, 2021

© 2021 Wiley-VCH GmbH

Order Completed

Thank you for your order.

This Agreement between Kai Zhang ("You") and John Wiley and Sons ("John Wiley and Sons") consists of your license details and the terms and conditions provided by John Wiley and Sons and Copyright Clearance Center.

Your confirmation email will contain your order number for future reference.

**License Number**	5931810533702
**License date**	Dec 18, 2024

Printable Details

Licensed Content

**Licensed Content Publisher**	John Wiley and Sons
**Licensed Content Publication**	Advanced Materials
**Licensed Content Title**	An Innovative Solvent-Responsive Coiling–Expanding Stent
**Licensed Content Author**	Shuo Shi, Miao Cui, Fengxin Sun, et al
**Licensed Content Date**	Jul 4, 2021
**Licensed Content Volume**	33
**Licensed Content Issue**	32
**Licensed Content Pages**	10

Order Details

**Type of use**	Journal/Magazine
**Requestor type**	University/Academic
**Is the reuse sponsored by or associated with a pharmaceutical or medical products company?**	no
**Format**	Electronic
**Portion**	Figure/table
**Number of figures/tables**	2
**Will you be translating?**	No
**Circulation**	500 - 999

About Your Work

**Title of new article**	Smart Materials Strategy for Vascular Challenges Targeting In-Stent Restenosis: A Critical Review
**Lead author**	Kai Zhang
**Title of targeted journal**	Regenerative Biomaterials
**Publisher**	Oxford University Press
**Expected publication date**	Feb 2025

Additional Data

**Portions**	Figure 6 and Figure 7
**The Requesting Person / Organization to Appear on the License**	Kai Zhang

Requestor Location

	Mr. Zhang Kai<br>China Jilin University
Requestor Location	
	Jilin,<br>China

Tax Details

Publisher Tax ID	EU826007151

Order Reference Number

Order reference number	Figure 3

Would you like to purchase the full text of this article? If so, please continue on to the content ordering system located here: Purchase PDF
If you click on the buttons below or close this window, you will not be able to return to the content ordering system.

Total: 0.00 USD

CLOSE WINDOW

ORDER MORE

© 2024 Copyright - All Rights Reserved | Copyright Clearance Center, Inc. | Privacy statement | Data Security and Privacy | For California Residents | Terms and ConditionsComments? We would like to hear from you. E-mail us at customercare@copyright.com

RightsLink

ZK

NIR Light-Triggered Shape Memory Polymers Based on Mussel-Inspired Iron–Catechol Complexes

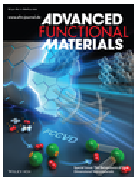

Author: Bilal Ul Amin, Nan Wang, Li Wang, et al
Publication: Advanced Functional Materials
Publisher: John Wiley and Sons
Date: Jun 4, 2021

© 2021 Wiley-VCH GmbH

Order Completed

Thank you for your order.

This Agreement between Kai Zhang ("You") and John Wiley and Sons ("John Wiley and Sons") consists of your license details and the terms and conditions provided by John Wiley and Sons and Copyright Clearance Center.

Your confirmation email will contain your order number for future reference.

**License Number**	5931810817493
**License date**	Dec 18, 2024

Printable Details

Licensed Content

**Licensed Content Publisher**	John Wiley and Sons
**Licensed Content Publication**	Advanced Functional Materials
**Licensed Content Title**	NIR Light-Triggered Shape Memory Polymers Based on Mussel-Inspired Iron–Catechol Complexes
**Licensed Content Author**	Bilal Ul Amin, Nan Wang, Li Wang, et al
**Licensed Content Date**	Jun 4, 2021
**Licensed Content Volume**	31
**Licensed Content Issue**	32
**Licensed Content Pages**	11

Order Details

**Type of use**	Journal/Magazine
**Requestor type**	University/Academic
**Is the reuse sponsored by or associated with a pharmaceutical or medical products company?**	no
**Format**	Electronic
**Portion**	Figure/table
**Number of figures/tables**	1
**Will you be translating?**	No
**Circulation**	500 - 999

About Your Work

**Title of new article**	Smart Materials Strategy for Vascular Challenges Targeting In-Stent Restenosis: A Critical Review
**Lead author**	Kai Zhang
**Title of targeted journal**	Regenerative Biomaterials
**Publisher**	Oxford University Press
**Expected publication date**	Feb 2025

Additional Data

**Portions**	Figure 4
**The Requesting Person / Organization to Appear on the License**	Kai Zhang

Requestor Location

	Mr. Zhang Kai<br>China Jilin University
**Requestor Location**	
	Jilin,<br>China

Tax Details

**Publisher Tax ID**	EU826007151

Order Reference Number

**Order reference number**	Figure 4

Would you like to purchase the full text of this article? If so, please continue on to the content ordering system located here: Purchase PDF
If you click on the buttons below or close this window, you will not be able to return to the content ordering system.

Total: 0.00 USD

CLOSE WINDOW

ORDER MORE

© 2024 Copyright - All Rights Reserved | Copyright Clearance Center, Inc. | Privacy statement | Data Security and Privacy | For California Residents | Terms and ConditionsComments? We would like to hear from you. E-mail us at customercare@copyright.com

RightsLink

Adaptive wireless millirobotic locomotion into distal vasculature

Author: Tianlu Wang et al

Publication: Nature Communications

Publisher: Springer Nature

Date: Aug 1, 2022

Copyright © 2022, The Author(s)

Creative Commons

This is an open access article distributed under the terms of the Creative Commons CC BY license, which permits unrestricted use, distribution, and reproduction in any medium, provided the original work is properly cited.

You are not required to obtain permission to reuse this article.
To request permission for a type of use not listed, please contact Springer Nature

© 2024 Copyright - All Rights Reserved | Copyright Clearance Center, Inc. | Privacy statement | Data Security and Privacy | For California Residents | Terms and ConditionsComments? We would like to hear from you. E-mail us at customercare@copyright.com

RightsLink

ZK

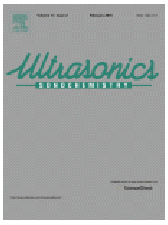

Targeting and deep-penetrating delivery strategy for stented coronary artery by magnetic guidance and ultrasound stimulation

Author:
Siyu Wang,Xixi Guo,Lili Ren,Bo Wang,Lixin Hou,Hao Zhou,Qinchang Gao,Yu Gao,Lianhui Wang

Publication: Ultrasonics Sonochemistry

Publisher: Elsevier

Date: October 2020

© 2020 Elsevier B.V. All rights reserved.

Order Completed

Thank you for your order.

This Agreement between Kai Zhang ("You") and Elsevier ("Elsevier") consists of your license details and the terms and conditions provided by Elsevier and Copyright Clearance Center.

Your confirmation email will contain your order number for future reference.

Printable Details

License Number	5931811262130
License date	Dec 18, 2024

Licensed Content

Licensed Content Publisher	Elsevier
Licensed Content Publication	Ultrasonics Sonochemistry
Licensed Content Title	Targeting and deep-penetrating delivery strategy for stented coronary artery by magnetic guidance and ultrasound stimulation
Licensed Content Author	Siyu Wang,Xixi Guo,Lili Ren,Bo Wang,Lixin Hou,Hao Zhou,Qinchang Gao,Yu Gao,Lianhui Wang
Licensed Content Date	Oct 1, 2020
Licensed Content Volume	67
Licensed Content Issue	n/a
Licensed Content Pages	1

Order Details

Type of Use	reuse in a journal/magazine
Requestor type	academic/educational institute
Portion	figures/tables/illustrations
Number of figures/tables/illustrations	2
Format	electronic
Are you the author of this Elsevier article?	No
Will you be translating?	No

About Your Work

Title of new article	Smart Materials Strategy for Vascular Challenges Targeting In-Stent Restenosis: A Critical Review
Lead author	Kai Zhang
Title of targeted journal	Regenerative Biomaterials
Publisher	Oxford University Press
Expected publication date	Feb 2025

Additional Data

Portions	Figure 1 and Figure 3
The Requesting Person / Organization to Appear on the License	Kai Zhang

Requestor Location

Mr. Zhang Kai
China Jilin University

Requestor Location

Jilin,
China

Tax Details

Publisher Tax ID GB 494 6272 12

Order Reference Number

Order reference number Figure 5

Total: 0.00 USD

CLOSE WINDOW

ORDER MORE

© 2024 Copyright - All Rights Reserved | Copyright Clearance Center, Inc. | Privacy statement | Data Security and Privacy | For California Residents | Terms and ConditionsComments? We would like to hear from you. E-mail us at customercare@copyright.com

RightsLink

ZK

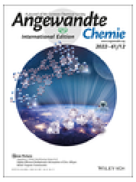

Ultrasound-Responsive Aqueous Two-Phase Microcapsules for On-Demand Drug Release

Author: Samuel K. Sia, Parag V. Chitnis, Xuanhe Zhao, et al

Publication: Angewandte Chemie International Edition

Publisher: John Wiley and Sons

Date: Mar 16, 2022

© 2022 Wiley-VCH GmbH

Order Completed

Thank you for your order.

This Agreement between Kai Zhang ("You") and John Wiley and Sons ("John Wiley and Sons") consists of your license details and the terms and conditions provided by John Wiley and Sons and Copyright Clearance Center.

Your confirmation email will contain your order number for future reference.

**License Number**	5931811475294
**License date**	Dec 18, 2024

Printable Details

Licensed Content

**Licensed Content Publisher**	John Wiley and Sons
**Licensed Content Publication**	Angewandte Chemie International Edition
**Licensed Content Title**	Ultrasound-Responsive Aqueous Two-Phase Microcapsules for On-Demand Drug Release
**Licensed Content Author**	Samuel K. Sia, Parag V. Chitnis, Xuanhe Zhao, et al
**Licensed Content Date**	Mar 16, 2022
**Licensed Content Volume**	61
**Licensed Content Issue**	20
**Licensed Content Pages**	7

Order Details

**Type of use**	Journal/Magazine
**Requestor type**	University/Academic
**Is the reuse sponsored by or associated with a pharmaceutical or medical products company?**	no
**Format**	Electronic
**Portion**	Figure/table
**Number of figures/tables**	1
**Will you be translating?**	No
**Circulation**	500 - 999

About Your Work

**Title of new article**	Smart Materials Strategy for Vascular Challenges Targeting In-Stent Restenosis: A Critical Review
**Lead author**	Kai Zhang
**Title of targeted journal**	Regenerative Biomaterials
**Publisher**	Oxford University Press
**Expected publication date**	Feb 2025

Additional Data

**Portions**	Figure 1
**The Requesting Person / Organization to Appear on the License**	Kai Zhang

Requestor Location

	Mr. Zhang Kai<br>China Jilin University
**Requestor Location**	
	Jilin,<br>China

Tax Details

**Publisher Tax ID**	EU826007151

Order Reference Number

**Order reference number**	Figure 6

Would you like to purchase the full text of this article? If so, please continue on to the content ordering system located here: Purchase PDF
If you click on the buttons below or close this window, you will not be able to return to the content ordering system.

Total: 0.00 USD

CLOSE WINDOW   ORDER MORE

© 2024 Copyright - All Rights Reserved | Copyright Clearance Center, Inc. | Privacy statement | Data Security and Privacy | For California Residents | Terms and ConditionsComments? We would like to hear from you. E-mail us at customercare@copyright.com

RightsLink

3D printed electro-responsive system with programmable drug release

Author:
Manal E. Alkahtani,Siyuan Sun,Christopher A.R. Chapman,Simon Gaisford,Mine Orlu,Moe Elbadawi,Abdul W. Basit

Publication: Materials Today Advances

Publisher: Elsevier

Date: August 2024

© 2024 The Authors. Published by Elsevier Ltd.

Creative Commons

This is an open access article distributed under the terms of the Creative Commons CC-BY license, which permits unrestricted use, distribution, and reproduction in any medium, provided the original work is properly cited.

You are not required to obtain permission to reuse this article.

To request permission for a type of use not listed, please contact Elsevier Global Rights Department.

Are you the author of this Elsevier journal article?

© 2024 Copyright - All Rights Reserved | Copyright Clearance Center, Inc. | Privacy statement | Data Security and Privacy | For California Residents | Terms and ConditionsComments? We would like to hear from you. E-mail us at customercare@copyright.com

MDPI
Journals Topics Information Editing Services Initiatives About
Sign In / Sign Up Submit
Search for Articles: Title / Keyword Author / Affiliation / Email Materials All Article Types Search Advanced

materials
Submit to this Journal
Review for this Journal
Propose a Special Issue

Order Article Reprints
Altmetric Share Help Cite Discuss in SciProfiles Endorse Comment

Open Access Article

3D-Printed PCL/PLA Composite Stents: Towards a New Solution to Cardiovascular Problems

by Antonio J. Guerra [1], Paula Cano [2], Marc Rabionet [1,2], Teresa Puig [2] and Joaquim Ciurana [1,*]

1 Department of Mechanical Engineering and Civil Construction, Universitat de Girona, C/Maria Aurèlia Capmany 61, 17003 Girona, Spain
2 Department of Medical Sciences, Faculty of Medicine, University of Girona, Emili Grahit 77, 17003 Girona, Spain
* Author to whom correspondence should be addressed.

Materials **2018**, *11*(9), 1679; https://doi.org/10.3390/ma11091679

Submission received: 31 July 2018 / Revised: 4 September 2018 / Accepted: 9 September 2018 / Published: 11 September 2018

(This article belongs to the Special Issue Biodegradable Polymeric Composites: Development and Industrial Applications)

Download Browse Figures Versions Notes

External Open Access Resources

Those who are new to the concept of open access might find the following websites or the *Open Access Explained!* video informative:

- **Wikipedia article on Open Access (https://en.wikipedia.org/wiki/Open_access)**
- **Open Access Network (https://open-access.network/en/information/open-access-primers/what-does-open-access-mean)**

Meaning of Open Access

In accordance with major definitions of open access in scientific literature (namely the Budapest, Berlin, and Bethesda declarations), MDPI defines *open access* by the following conditions:

- peer-reviewed literature is freely available without subscription or price barriers,
- literature is immediately released in open access format (no embargo period), and
- published material can be re-used without obtaining permission as long as a correct citation to the original publication is given.

Open Access Explained!

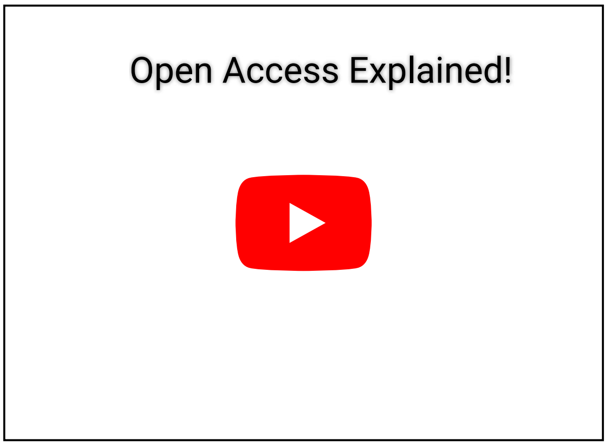

Until 2008, most articles published by MDPI contained the note: "© *year* by MDPI (http://www.mdpi.org). Reproduction is permitted for noncommercial purposes". During 2008, MDPI journals started to publish articles under the **Creative Commons Attribution License (http://creativecommons.org/licenses/by/4.0/)** and are now using the latest version of the CC BY license, which grants authors the most extensive rights. All articles published by MDPI before and during 2008 should now be considered as having been released under the post-2008 Creative Commons Attribution License.

This means that all articles published in MDPI journals, including data, graphics, and supplements, can be linked from external sources, scanned by search engines, re-used by text mining applications or websites, blogs, *etc.* free of charge under the sole condition of proper accreditation of the source and original publisher. MDPI believes that open access publishing fosters the exchange of research results amongst scientists from different disciplines, thus facilitating interdisciplinary research. Open access publishing also provides access to research results to researchers worldwide, including those from developing countries, and to an interested general public. Although MDPI publishes all of its journals under the open access model, we believe that open access is an enriching part of the scholarly communication process that can and should co-exist with other forms of communication and publication, such as society-based publishing and conferencing activities.

Important Note: some articles (especially *Reviews*) may contain figures, tables or text taken from other publications, for which MDPI does not hold the copyright or the right to re-license the published material. Please note that you should inquire with the original copyright holder (usually the original publisher or authors), whether or not this material can be re-used.

Advantages of Open Access for Authors

The High Availability and Visibility of our open access articles is guaranteed through the free and unlimited accessibility of the publication over the Internet. Everyone can freely access and download the full text of all articles published with MDPI: readers of open access journals, *i.e.*, mostly other researchers, do not need to pay any subscription or pay-per-view charges to read articles published by MDPI. Open access publications are also more likely to be included in search engines and indexing databases.

The Higher Citation Impact of open access articles results from their high publicity and availability. Open access publications are demonstrably more frequently cited [1,2].

Lower Publishing Costs: Open access publishers cover their costs for editorial handling and editing of a paper by charging authors' institutes or research funding agencies. The cost of handling and producing an article is covered through the one-time payment of an **article processing charge (APC) (/about/apc)** for each accepted article. The APCs of open access publishers are only a fraction of the average income per paper earned by traditional, subscription-based publishers. MDPI's **article processing charge (APC) (/about/apc)** is the same, irrespective of article length, because we wish to encourage publication of long papers with complete results and full experimental or computational details [3].

Faster Publication in MDPI's open access journals is achieved by online-only availability. Accepted articles are typically published online more rapidly in MDPI journals than those of traditional, subscription-based and printed journals are [4].

Links and Notes

1. Open access citation impact advantage: **http://en.wikipedia.org/wiki/Open_access#Authors_and_researchers (https://en.wikipedia.org/wiki/Open_access_(publishing)#Authors_and_researchers)**. For example, a standard research paper "Shutalev, A.D.; Kishko, E.A.; Sivova, N.V.; Kuznetsov, A.Y. *Molecules* **1998**, *3*, 100-106" has been cited 51 times, the highest number among all the papers published so far by the same author.
2. Lin, S.-K. *Editorial:* Non-Open Access and Its Adverse Impact on Molecules. *Molecules* **2007**, *12*, 1436-1437 (PDF format 16 K, HTML format).
3. A research paper of 30 pages has been published: ***Molecules* 2008, *13*(5), 1081-1110 (/1420-3049/13/5/1081)**.
